# Proliferation, migration and phenotypic transformation of VSMC induced via Hcy related to up-expression of WWP2 and p-STAT3

**DOI:** 10.1371/journal.pone.0296359

**Published:** 2024-01-02

**Authors:** Xiuyu Wang, Na Gui, Xing Ma, Yue Zeng, Tingrun Mo, Minghao Zhang

**Affiliations:** 1 Department of Pathophysiology, School of Basic Medical Sciences, Ningxia Medical University, Yinchuan, Ningxia, P.R. China; 2 Key Laboratory of Metabolic Cardiovascular Diseases Research of National Health Commission, Ningxia Key Laboratory of Vascular Injury and Repair Research, Yinchuan, Ningxia, P.R. China; Army Medical University, CHINA

## Abstract

To provide a theoretical basis for the prevention and treatment of atherosclerosis (AS), the current study aimed to investigate the mechanism underlying the effect of homocysteine (Hcy) on regulating the proliferation, migration and phenotypic transformation of vascular smooth muscle cells (VSMC) via sirtuin-1 (SIRT1)/signal transducer and activator of transcription 3 (STAT3) through Nedd4-like E3 ubiquitin-protein ligase WWP2 (WWP2). Here, Based on the establishment of ApoE^-/-^ mouse models of high Hcy As and the model of Hcy stimulation of VSMC *in vitro* to observe the interaction between WWP2 and STAT3 and its effect on the proliferation, migration, and phenotypic transformation of Hcy-induced VSMC, which has not been previously reported. This study revealed that WWP2 could promote the proliferation, migration, and phenotype switch of Hcy-induced VSMC by up-regulating the phosphorylation of SIRT1/STAT3 signaling. Furthermore, Hcy might up-regulate WWP2 expression by inhibiting histone H3K27me3 expression through up-regulated UTX. These data suggest that WWP2 is a novel and important regulator of Hcy-induced VSMC proliferation, migration, and phenotypic transformation.

## Introduction

Atherosclerosis (AS), arising from multiple factors, such as abnormal lipid metabolism and uncontrolled immune responses, is a chronic inflammatory disease commonly affecting vascular smooth muscle cells (VSMC). VSMC, located in the tunica media of arteries, play a significant role in the formation of AS [1]. Additionally, it has been reported that the proliferation, migration and phenotypic switch of VSMC serve a critical effect on vascular diseases. Therefore, a previous study demonstrated that angiotensin II (AngII) could bind to AngII type 1 receptor on VSMC to activate signal transducer and activator of transcription 3 (STAT3), which in turn promoted the proliferation and migration of VSMC, thus representing the key events in hypertension-induced vascular lesions [2]. Based on their structural and functional differences, VSMC can be divided into those showing a contractile and those exhibiting a synthetic phenotype. The main function of contractile VSMC is to contract and maintain the vascular wall tension, while synthetic VSMC are characterized by the large amount of organelles and small number of muscle fibers, thus promoting VSMC proliferation and migration [3]. Under normal conditions, VSMC exhibit a contractile phenotype, which is involved in fighting vascular tension and maintaining vascular wall homeostasis. However, in several pathological states, VSMC experience transformation to a synthetic phenotype, thus enhancing VSMC proliferation and migration, and excessive extracellular matrix synthesis. The above changes can lead to increased lipid phagocytosis of smooth muscle cells and their transport into the intima of the arteries, eventually promoting the transformation of VSMC to foam cells [4]. A study showed that the AngII-mediated activation of STAT3 could promote the transition of VSMC from a contractile to a synthetic phenotype, thus supporting the effect of VSMC on hypertension-induced angiopathy [5]. The transformation of smooth muscle cells from a contractile to a synthetic phenotype serves a significant role in the progression of AS [6]. Although several studies have been conducted on the phenotypic transition of VSMC, the specific molecular mechanism underlying their phenotypic switch remains unclear.

Hyperhomocysteinemia (HHcy), an independent risk factor for AS, is no less harmful than hyperlipidemia. It has been reported that homocysteine (Hcy) can enhance the proliferation and migration of VSMC, and induce them to secrete proteases, such as matrix metalloproteinases, which affect the dynamic balance of extracellular matrix and promote the phenotypic transformation of VSMC [7]. Although it has been previously demonstrated that Hcy can induce the proliferation, migration and phenotypic switch of VSMC [8], its detailed underlying mechanism of action remains elusive. Given the role of these changes in the development of AS, understanding the molecular mechanisms underlying the changes in Hcy-induced VSMC is of great significance.

Signal transducer and activator of transcription 3 (STAT3) serves a significant effect on the regulation of VSMC. Therefore, a previous study suggested that the phosphorylation of STAT3 could be involved in the proliferation, migration and phenotypic transformation of VSMC [9]. STAT3 activation is commonly triggered by its phosphorylation, which is regulated by deacetylase NAD-dependent deacetylase sirtuin-1 (SIRT1) [10–12]. It has been reported that SIRT1 deacetylates STAT3, thus blocking STAT3 phosphorylation, eventually inhibiting the AngII-induced proliferation, migration and phenotypic transformation of VSMC [11, 12].

Nedd4-like E3 ubiquitin-protein ligase WWP2 (WWP2), an E3 ubiquitin-protein ligase of the HTECT-type NEDD4 family, interacts with different substrates to improve diabetes, pathological myocardial fibrosis and heart failure. Therefore, WWP2 could be a potential target for the targeted therapy of several cardiovascular system-related diseases [13]. However, whether WWP2 is involved in the physiological and pathological processes of VSMC remains unknown. Therefore, investigating the role and the molecular mechanism underlying the effect of WWP2 on the phenotypic transformation of VSMC, as well as on their proliferation and migration abilities, could provide novel insights into the treatment of atherosclerotic vascular diseases, such as stroke, ischemic cardiomyopathy and heart failure. A previous study showed that WWP2 was upregulated in AngII-induced VSMC and promoted their phenotypic switch via regulating STAT3 phosphorylation through SIRT1 [14]. Additionally, WWP2 could modulate hypertensive angiopathy via regulating SIRT1/STAT3 pathway, while WWP2 knockdown in VSMC could alleviate hypertensive angiopathy both *in vitro* and *in vivo*. Therefore, previous studies revealed that WWP2 could form a complex with STAT3/SIRT1 to abrogate the inhibitory effect of SIRT1 on STAT3, thus enhancing STAT3-K685 acetylation and STAT3-Y705 phosphorylation. In turn, the above process could promote the AngII-mediated proliferation, migration and phenotypic transformation of VSMC and the development of hypertensive angiopathy *in vivo* [13, 14]. This finding indicated that the SIRT1-dependent STAT3 phosphorylation could exhibit a critical role in the proliferation, migration and phenotypic transformation of VSMC, and this effect may be regulated by WWP2. However, whether the Hcy-induced proliferation, migration and phenotypic transformation of VSMC is triggered by a similar mechanism has not been previously investigated.

The current study aimed to uncover the association between WWP2 and SIRT1/STAT3 signaling and their effects on the proliferation, migration and phenotypic transformation of Hcy-induced VSMC both *in vivo* and *in vitro*. Therefore, a high Hcy apolipoprotein E deficient (ApoE^-/-^) mouse model and a Hcy-stimulated VSMC *in vitro* model were established. More specifically, the current study explored whether WWP2 could serve a functional role in Hcy-induced VSMC proliferation, migration and phenotypic transformation, possibly via regulating SIRT1/STAT3 phosphorylation. This finding could provide a significant experimental and research basis for studying the pathogenesis of Hcy-mediated AS.

## Materials and methods

### Animal treatment

Six weeks old male ApoE^-/-^ mice (weight, 25–28 g) were purchased from Beijing Vital River Laboratory Animal Technology Co., Ltd. and were then randomly divided into the following two groups (n = 6/group): i. ApoE^-/-^ mice fed with normal diet (ApoE^-/-^ + NC group); and ii. ApoE^-/-^ mice fed with 1.7% methionine diet (ApoE^-/-^ + HMD group). The mice were kept constant at 22°C, the humidity was 60%, the light and dark alternated for 12h each, and they were given drinking water and corresponding feed every day, and their weight and physiological state were recorded every week. At 24 weeks, all mice were fasted overnight and anesthetized by intraperitoneal injection of pentobarbital sodium (50 mg/kg body weight, Shenzhen Haiwang Pharmaceutical Co., Ltd.). At the end of the experiment, 5% isoflurane was used for euthanasia. Death was confirmed when mice developed respiratory arrest. Blood was collected from the orbital sinus and centrifuged at 3,000 rpm for 15 min at 4˚C. The serum levels of Hcy, total cholesterol (TC) and triglyceride (TG) were measured using an automatic biochemical analyzer (Beckman Coulter) as previously described [15, 16]. In addition, aortas were isolated from the heart of mice, frozen in liquid nitrogen and stored at -80˚C until further use. The study was approved by the Ethics Committee of Ningxia Medical University (NO. 2023–038) and conducted by the Guide for the Care and Use of Laboratory Animals, and the reporting follows the recommendations in the ARRIVE guidelines.

### Immunofluorescence staining

Immunofluorescence staining of the frozen aortic root sections (5.0-μm thick) from ApoE^-/-^ mice was performed as previously described. Briefly, sections were fixed in cold acetone for 20 min, blocked with goat serum and stained with primary antibodies against α-SMA, SM22α, OPN, SIRT1 and p-STAT3 (all from Abcam) at 4˚C overnight. Images were captured under a laser scanning confocal microscopy and colocalization analysis was performed using Coloc 2 plugin in ImageJ [17].

### Cell transfection

The sequences of WWP2 overexpression plasmid, GFP, Si-NC, and Si-WWP2 were obtained from Genepharma (Shanghai, China) and infected as previously described [18]. And they were transiently transfected into the cells using Lipofectamine 2000 (Life Technologies, Gaithersburg, MD, USA) following the manufacturer’s instruction. The transfection efficiency was detected by qRT-PCR and western blot, then the cells were collected for downstream analysis.

### Cell culture and treatment

Human VSMC were purchased from the BeNa Culture Collection (BNCC; Suzhou Bena Chuanglian Biotechnology Co. Ltd.) and cultured in DMEM (Gibco, Grand Island, NY, USA) supplemented with 7% fetal bovine serum (FBS; Gibco; Thermo Fisher Scientific, Inc.) and 1% penicillin/streptomycin solution (Beijing Solarbio Science & Technology Co., Ltd.) at 37˚C in an incubator with 5% CO_2_. VSMC were divided into the normal control, Hcy, Hcy+WWP2, GFP, Si-NC, Hcy+Si-WWP2, Hcy+Ex527 (SIRT1 inhibitor; Beyotime Institute of Biotechnology), Hcy+ SRT1720 (SIRT1 agonist; Beyotime Institute of Biotechnology), Hcy+Si-WWP2+SRT1720 and Hcy+Si-WWP2+Ex527 groups. All cells used were between passages 3 and 7. Prior each experiment VSMC were induced with 100 μmol/l Hcy for 48 h.

### MTT assay

The proliferation ability of VSMC was assessed using a MTT assay. Briefly, a total of 5x10^3^ cells/well from each group (control, Hcy, Hcy+Si-WWP2, and Hcy+WWP2 groups) were seeded into 96-well plates and allowed to attach in DMEM with 7% FBS for 48 h. Cells were then exposed to various treatments and incubated at 37˚C in an incubator with 5% CO_2_ for 48 h. Subsequently, 50 μl MTT (Nanjing KeyGen Biotech, Co., Ltd.) solution was added into each well and cells were incubated at 37˚C for an additional 4 h. Finally, each well was supplemented with 150 μl DMSO (Beijing Solarbio Science & Technology Co., Ltd.) and the absorbance at a wavelength of 570 nm was measured using a spectrophotometer (BioTek Epoch; BioTek Instruments, Inc.). All values were normalized to those of the control group [8].

### EdU assay

The proliferation ability of VSMC was assessed by EdU assay. Briefly, cells in the logarithmic growth phase were digested with trypsin. Subsequently, VSMC from each group (control, Hcy, Hcy+Si-WWP2, and Hcy+WWP2groups)were cultured in DMEM supplemented with 7% FBS at a density of 5×10^3^ cells/well, seeded onto laser confocal dishes and allowed to attach for 48 h. The cells were then supplemented with 50 μM EdU (Guangzhou RiboBio Co., Ltd.), followed by incubation for an additional 2 h. Cells were then washed with PBS, fixed with 4% cold formaldehyde for 30 min and incubated with glycine for 5 min. After permeabilization with 0.5% TritonX-100 for 10 min, cells were treated with 1x Apollo staining reaction solution for 30 min. Subsequently, cells were washed twice with 0.5% TritonX-100 and the DNA content was stained with Hoechst 33342 for 30 min in the dark. EdU-labeled cells were counted under a fluorescent positive microscope (Leica Microsystems GmbH) and were normalized to the total number of Hoechst-positive cells [8].

### Wound healing assay

A wound healing assay was carried out to evaluate the cell migration ability of Hcy-treated VSMC. Briefly, 5×10^3^ VSMC in a volume of 100 μl/well were seeded in 6-well plates and were allowed to reach 80–90% confluency. Subsequently, a linear scratch wound was made at the center of the cell monolayer using a 200-μl tip. Following incubation for 0h and 48 h, images of the migrated cells were captured and their number was calculated under an image acquisition system microscope (Olympus Corporation) [8].

### Reverse transcription-quantitative PCR (RT-Qpcr)

To determine the Mrna expression levels, RT-Qpcr was performed. Total RNA was extracted from VSMC using the RNA simple Total RNA kit (Tiangen Biotech Co., Ltd.), according to the manufacturer’s instructions. Subsequently, 1 μg total RNA was reverse transcribed into Cdna using Cdna Reverse Transcription kits (Thermo Fisher. Scientific, Inc.) The mRNA expression levels of WWP2, UTX, SIRT1, α-SMA, SM22a and OPN were detected by qPCR using a SYBR green PCR Kit (DBI Bioscience) on the LightCycler System (Roche Applied Science). The primer sequences used are listed in Table I in S1 Table. The thermocycler conditions were as follows: 37˚C for 30 sec; 95˚C for 5 min; followed by 45 cycles of 95˚C for 10 sec; 55˚C for 30 sec; and 72˚C for 30 sec. The obtained Ct values were analyzed based on the amplification curves and the relative expression levels of the target genes were calculated using the 2^-ΔΔCq^ method [19]. PCR reactions were performed in triplicate and normalized using GAPDH as a reference gene.

### Western blot analysis

To detect the changes in the protein levels in VSMC, western blot analysis was carried out using specific antibodies [8, 20]. Total proteins were isolated from cells using a Whole protein extraction kit (Nanjing KeyGen Biotech Co., Ltd.), while protein concentration was determined using the SimpliNano™ Biochrom Spectrophotometer (Biochrom, Ltd.). The protein samples (20 μl/lane) were separated by SDS-PAGE and were then transferred onto a PVDF membrane (MilliporeSigma). Following blocking with 5% non-fat milk in PBS with Tween-20, the membrane was incubated at 4˚C overnight with the following antibodies: anti-α-SMA (dilution, 1:1,000, no.ab21027), anti-SM22a (dilution, 1:1,000, no.ab14106), anti-OPN (dilution, 1:1,000, no.ab214050), anti-WWP2 (dilution, 1:200, no.ab103527), anti-SIRT1 (dilution, 1:500, no.ab189494), anti-STAT3 (1:800, no. ab68153.), anti-phospho (p)**-**STAT3 (1:300, no. ab117253.), anti-UTX (1:1200, no. ab36938.), anti-H3K27me3 (1:1000, no. ab1791.), and anti-β-actin (dilution, 1:1,000, no.ab8227. all from Abcom.). Following washing, the membranes were incubated with the corresponding horseradish peroxidase-conjugated IgG (anti-rabbit, no. ZB2301 or anti-mouse, no. ZB2305, dilution, 1:5,000. ZSGB-BIO) for 4 h. Finally, the protein bands were visualized using chemiluminescence (ECL; Nanjing KeyGen Biotech Co., Ltd.).

### Statistical analyses

All statistical analyses were performed using GraphPad Prism 5.0 software. Data are expressed as the mean ± SD of at least three independent experiments. The differences of single parameters between two groups shuould be compared using unpaired Student’s t-test. while those among multiple groups using Kruskal-Wallis one-way ANOVA, followed by Dunn’s test. *P*≤0.05 was considered to indicate a statistically significant difference.

## Results

### Hcy promotes the transition of mouse aortic VSMC from a contractile to a secretory phenotype

**Fig 1:**
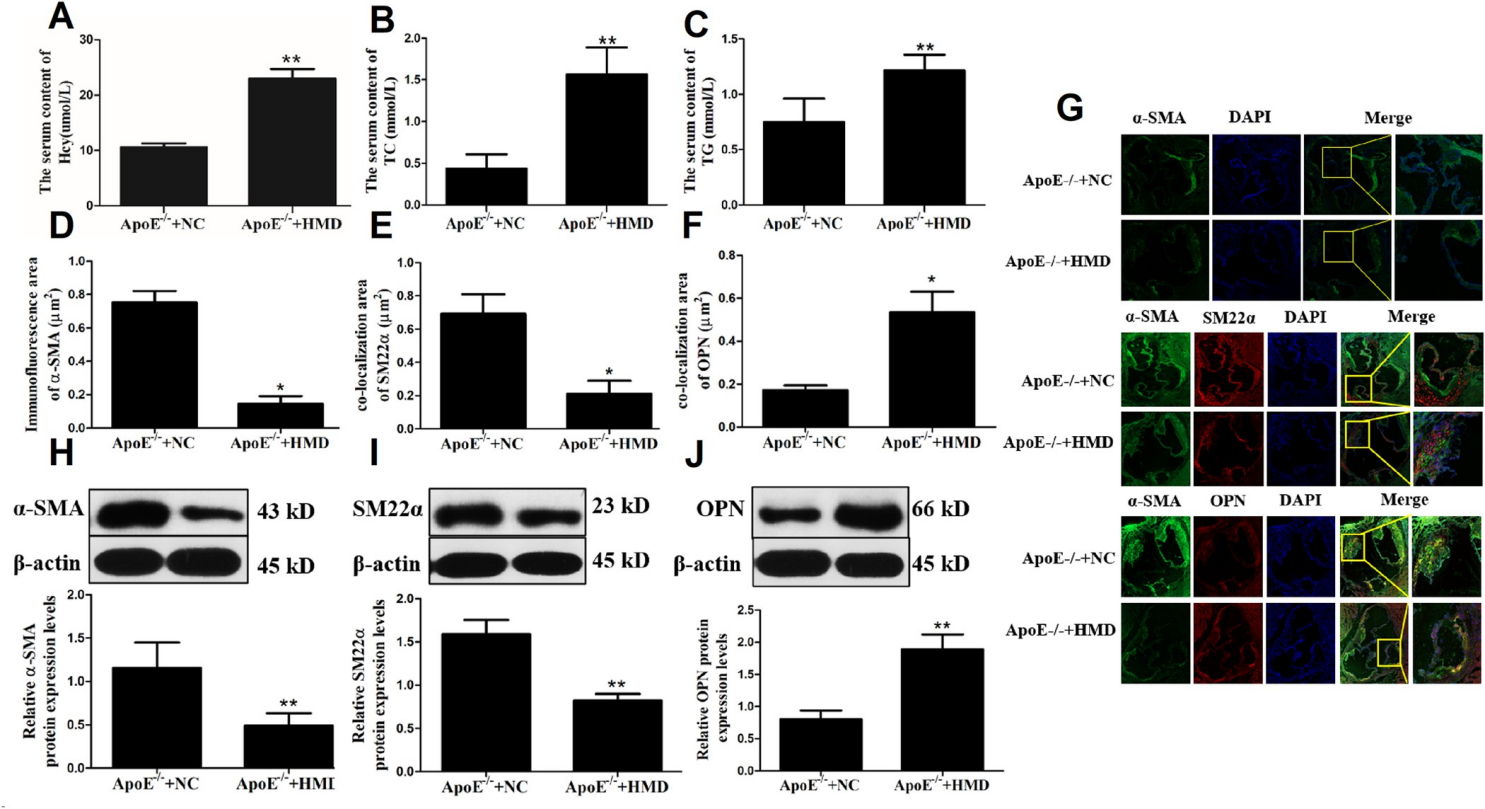
Hcy promoted the transformation of mice aortic VSMC from contractile to secretory phenotype. A-C. Changes of serum Hcy, TC and TG levels. D. Immunofluorescence staining methods were used to detect the α-SMA protein levels in ApoE^-/-^ mice aortic root frozen sections. E-F. Co-localization of α-SMA with SM22a and OPN in mice aortic VSMC, respectively. G. Immunofluorescence staining. The photographs are representative of three separate experiments. Blue fluorescence is the nucleus of the vascular smooth muscle cell (DAPI), and green and red fluorescence is the target protein (40×). H-J. Western blot analysis was used to detect the α-SMA, SM22a and OPN protein levels in ApoE^-/-^ mice aortic VSMC. The experiment was performed in triplicate, and the representative images are shown. *P<0.05, **P<0.01. compared with the ApoE^-/-^+NC group.

To verify that the ApoE^-/-^ HHcy mouse model was successfully established, the serum levels of Hcy, TC and TG were measured by an automatic biochemical analyzer (Fig 1A–1C). The results showed that the serum levels of Hcy exceeded 20 μmol/l, while those of TC and TG were significantly higher compared with the control group, thus verifying that the HHcy animal model was successfully replicated. Furthermore, to evaluate the effect of Hcy on the phenotype of VSMC, the protein expression levels of α-SMA, SM22α and OPN were detected in aortic root frozen sections of ApoE^-/-^ mice by immunofluorescence staining (Fig 1D–1G) and western blot analysis (Fig 1H–1J). α-SMA and SM22α are markers associated with contractile phenotype, while OPN with synthetic phenotype. Therefore, compared with the ApoE^-/-^ + NC group, the immunofluorescence staining area of α-SMA and SM22α in aortic VSMC was significantly reduced in the ApoE^-/-^ + HMD group, while that of OPN was notably increased,suggesting that the protein expression of α-SMA and SM22α decreased, while the protein expression of OPN increased. Meanwhile, western blot analysis also obtained the same results. These results indicated that treatment of aortic VSMC from ApoE^-/-^ mice with high concentrations of Hcy promoted their transformation from a contractile to a secretory phenotype.

### Hcy upregulates WWP2 in VSMC

**Fig 2:**
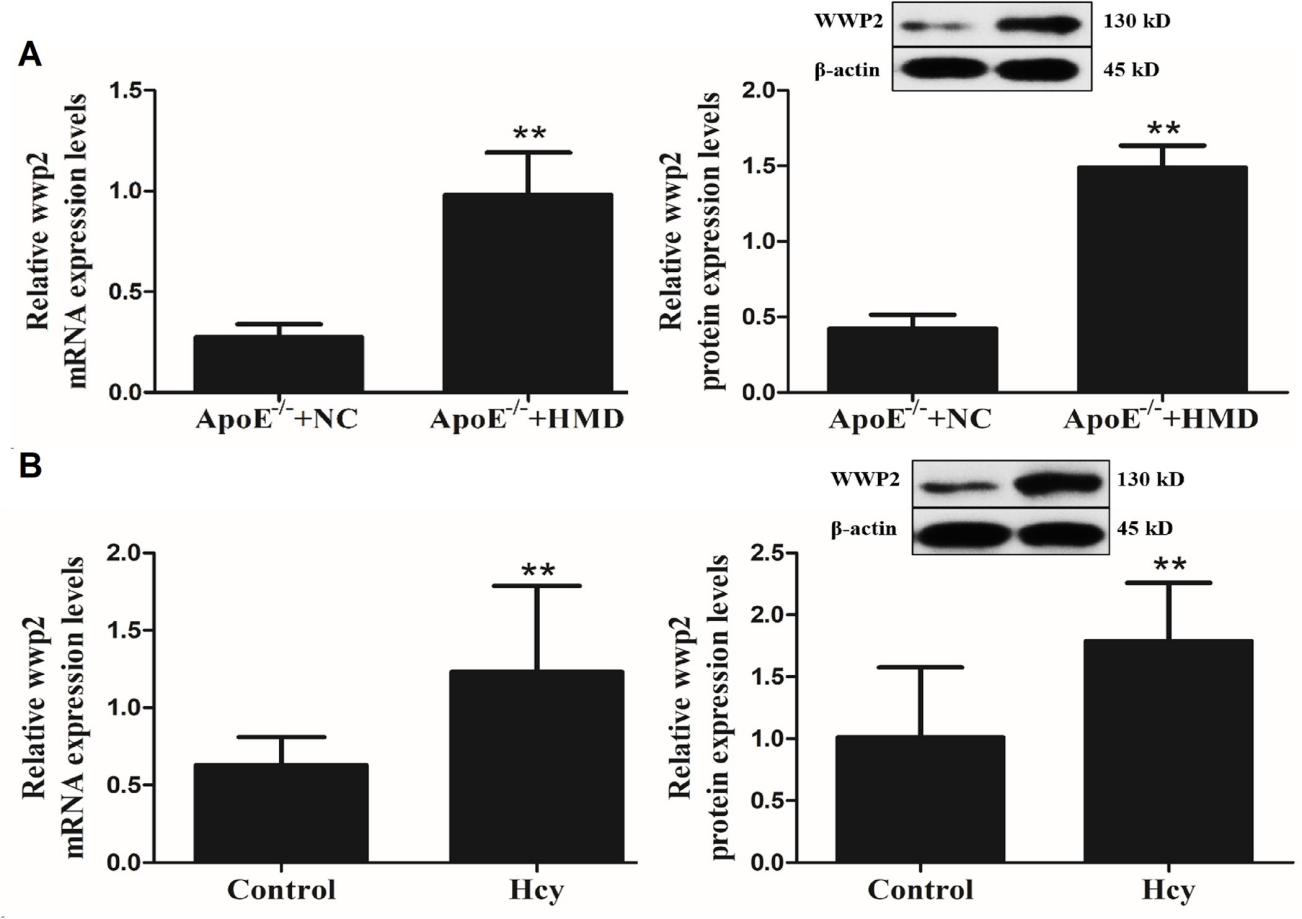
Hcy increased the expression of WWP2 in VSMC *in vivo and in vitro*. A. WWP2 mRNA and protein expression in mice aortic VSMC. B. WWP2 mRNA and protein expression in VSMC. The experiment was performed in triplicate, and the representative images are shown.**P<0.05. compared with the ApoE^-/-^+NC group and control group.

To investigate whether WWP2 was involved in the phenotypic transformation of VSMC in ApoE^-/-^ mice treated with Hcy, RT-qPCR and western blot analyses were performed to detect the mRNA and protein expression levels of WWP2 both *in vivo* and *in vitro* (Fig 2A and 2B). The results demonstrated that WWP2 was significantly upregulated by Hcy, thus suggesting that the Hcy-mediated injury of VSMC could be associated with increased WWP2 expression.

### WWP2 promotes the Hcy-induced proliferation, migration and phenotypic transformation of VSMC

**Fig 3:**
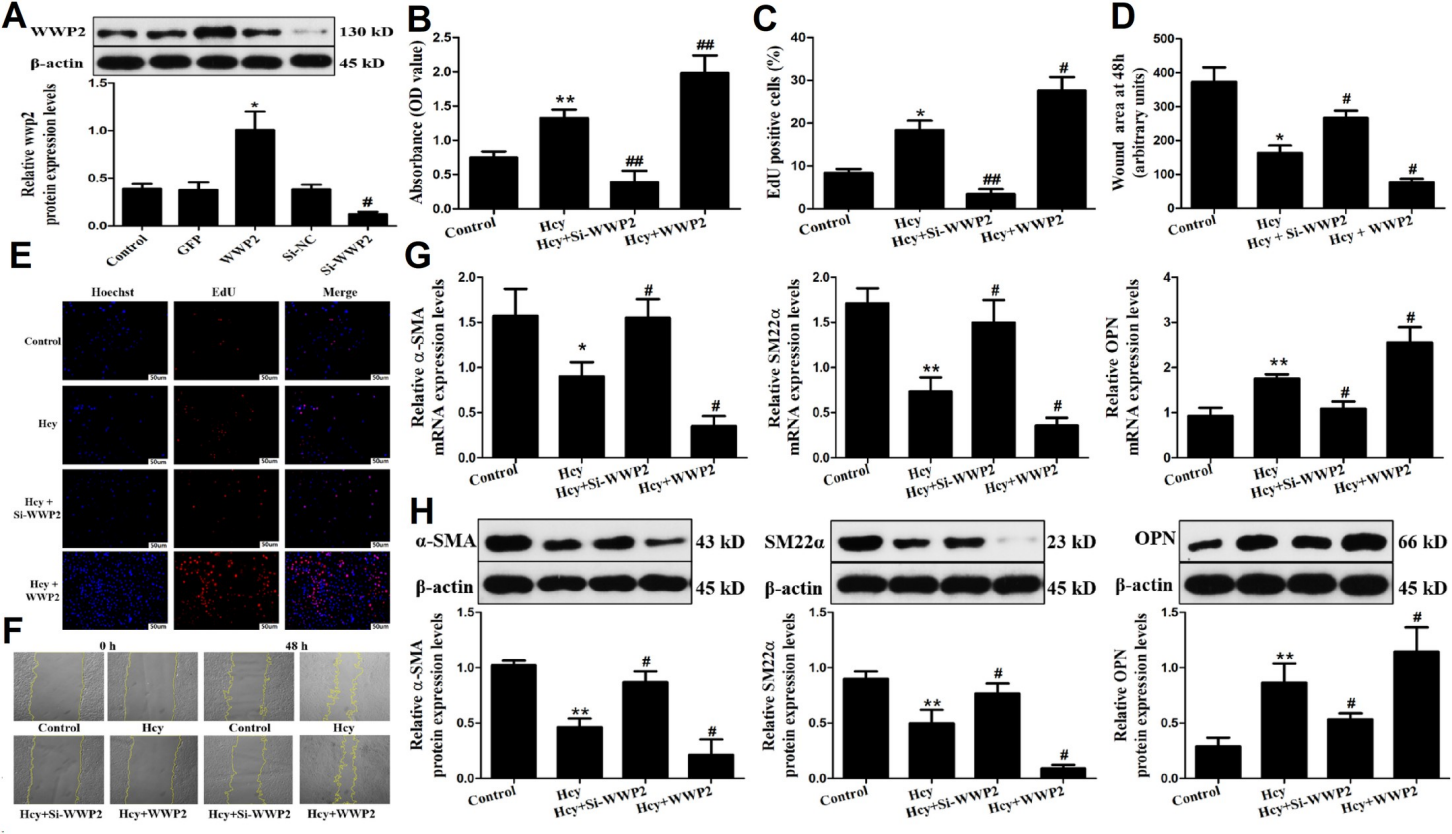
Overexpression of WWP2 promoted Hcy-induced proliferation, migration, and phenotypic transformation of VSMC. A. The transfection efficiency of WWP2 overexpression and interference plasmid was determined by western blot. B. Cell viability assessed using MTT assay. C. Quantification of results from EdU assays. D. Results of the scratch test. E. EdU staining diagram. Red fluorescence refers to nuclei in the proliferative period and blue fluorescence refers to nuclei in the non-proliferative period. The cell proliferation rate for each group was calculated by dividing the number of red nuclei by blue nuclei. F. Cell scratch test at 0h and 48h. G. qRT-PCR was used to detect the mRNA expressions of a-SMA, SM22a and OPN. H. Western blot was used to detect the protein expressions of a-SMA, SM22a and OPN. The experiment was performed in triplicate, and the representative images are shown.*P<0.05, **P<0.01 compared with the control group. ^**#**^ *P*<0.05, ^**##**^ *P*<0.01 compared with the Hcy group.

To assess the effect of WWP2 on promoting the Hcy-mediated VSMC proliferation, WWP2 was overexpressed or knocked down following VSMC transfection with the corresponding plasmids. Therefore, western blot analysis was performed to verify the transfection efficiency of VSMC with WWP2 or Si-WWP2 (Fig 3A). The results showed that WWP2 protein expression increased after transfection and treatment of WWP2 overexpression, while the expression of WWP2 interfering plasmid decreased, suggesting that WWP2 overexpression and interfering plasmid were successfully constructed. Subsequently, MTT (Fig 3B) and EdU (Fig 3C and 3E) assays were carried out to detect the changes in the proliferation ability of VSMC transfected with WWP2 or Si-WWP2. WWP2 overexpression significantly enhanced the proliferation ability of VSMC compared with the Hcy group. By contrast, cell proliferation was notably attenuated in Hcy-treated WWP2-depleted VSMC. The aforementioned results indicated that WWP2 could promote the proliferation of Hcy-induced VSMC.

It has been reported that the migration of VSMC from media to the subintima of the vascular wall is closely associated with the occurrence and development of AS [1]. Therefore, to reveal the role of WWP2 in the Hcy-induced migration ability of VSMC, a wound healing assay was performed and the migration rate of VSMC was determined after 48 h (Fig 3D and 3F). The results suggested that WWP2 could regulate the migration ability of Hcy-induced VSMC. Furthermore, to verify the effect of WWP2 on the Hcy-mediated phenotypic transformation of VSMC, cells transfected with WWP2 or Si-WWP2 were exposed to Hcy for 48 h. Then, the mRNA and protein expression levels of systolic phenotype-associated (α-SMA and SM22α) and synthetic phenotype-associated (OPN) genes were determined by RT-qPCR (Fig 3G) and western blot analysis (Fig 3H), respectively. The results showed that WWP2 overexpression further downregulated α-SMA and SM22α, and upregulated OPN compared with the Hcy group, both in the mRNA and protein levels. Together, the above results suggested that WWP2 overexpression could promote the Hcy-mediated transformation of VSMC from a contractile to a synthetic phenotype and enhance VSMC proliferation and migration.

### WWP2 inhibits SIRT1 and enhances STAT3 phosphorylation

**Fig 4:**
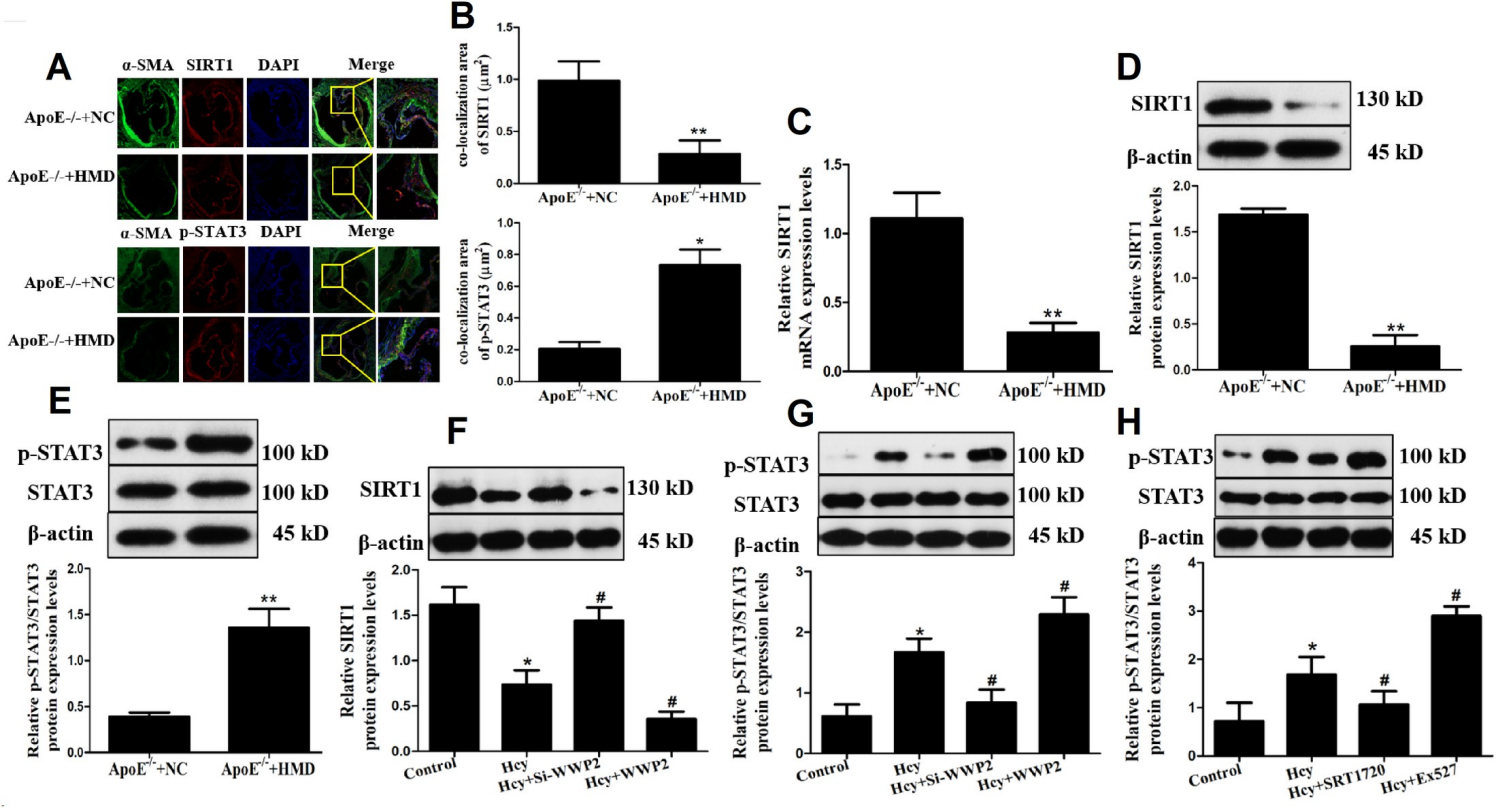
WWP2 inhibited SIRT1 and up-regulated STAT3 phosphorylation. A. Immunofluorescence staining. The photographs are representative of three separate experiments. Blue fluorescence is the nucleus of the vascular smooth muscle cell (DAPI), and green and red fluorescence is the target protein (40×). B. Co-localization of α-SMA with SIRT1 and p-STAT3 in mice aortic VSMC, respectively. C. SIRT1 mRNA expression in mice aortic VSMC. D. SIRT1 protein expression in mice aortic VSMC. E. p-STAT3 protein expression in mice aortic VSMC. F. SIRT1 protein expression in Hcy-induced VSMC. G. p-STAT3 and STAT3 protein expression in Hcy-induced VSMC. H. The effects of the Ex527 and SRT1720 on p-STAT3 and STAT3 protein expression in Hcy-induced VSMC. The experiment was performed in triplicate, and the representative images are shown.*P<0.05, **P<0.01 compared with the control group. ^#^*P*<0.05. compared with the Hcy group.

To further reveal the specific mechanism underlying the effect of WWP2 upregulation on Hcy-induced VSMC proliferation, migration and phenotypic transformation, α-SMA was co-located with SIRT1 and p-STAT3 to detect the expression of SIRT1 and p-STAT3 in VSMC. (Fig 4A and 4B). The results showed that both SIRT1 and p-STAT3 were expressed in ApoE^-/-^ mice aortic root vessels, suggesting that SIRT1-STAT3 signaling pathway was activated in VSMC. Aafter ImageJ analysis, compared with the control group, the co-localization area of SIRT1 in aorta VSMC of ApoE^-/-^+HMD group was significantly reduced, while the co-localization area of p-STAT3 was significantly increased. The results indicated that the protein expression of SIRT1 decreased and the protein expression of p-STAT3 increased. Meanwhile, the mRNA and protein expression of SIRT1 in mouse aorta decreased, while the protein expression of p-STAT3 increased (Fig 4C–4E). These results illustrated that Hcy stimulation promoted the activation of SIRT1-STAT3 signaling pathway in mouse aorta VSMC.

Furthermore, WWP2 overexpression plasmid transfected cells were treated with Hcy for 48 h. Subsequently, western blot analysis was carried out to detect the protein expression levels of SIRT1, STAT3 and p-STAT3. The results illustrated that Hcy could induce the expression of the SIRT1/STAT3 signaling pathway-related proteins in VSMC (Fig 4F, 4G). Compared with the Hcy group, SIRT1 was further downregulated and p-STAT3 was significantly upregulated in the Hcy + WWP2 group. By contrast, the protein expression levels of SIRT1 were increased, while those of p-STAT3 were decreased in the Hcy + Si-WWP2 group compared with the Hcy group. These findings indicated that the SIRT1/STAT3 signaling pathway could play a significant role in Hcy-induced VSMC injury and WWP2 could upregulate SIRT1 and attenuate STAT3 phosphorylation.

To explore whether the effects of Hcy on VSMC were dependent on the SIRT1 signaling pathway, VSMC, prior treatment with Hcy, were co-treated with Ex527, an SIRT1 signaling pathway inhibitor, for 48 h. In the Hcy + SRT1720 group, VSMC were treated with the SIRT1 signaling pathway stimulant SRT1720 for 2 h prior co-treatment with Hcy for 48 h. As shown in Fig 4H the protein expression levels of p-STAT3 were further elevated in the Hcy + EX527 group, while p-STAT3 was notably downregulated in the Hcy + SRT1720 group. Taken together, the above findings indicated that WWP2 could inhibit SIRT1 and enhance STAT3 phosphorylation, thus further suggesting that Hcy could regulate the SIRT1-mediated STAT3 phosphorylation via upregulating WWP2 to promote VSMC proliferation, migration and phenotypic switch.

### Hcy up-regulates WWP2 expression by inhibiting histone H3K27me3 via up-regulated UTX

**Fig 5:**
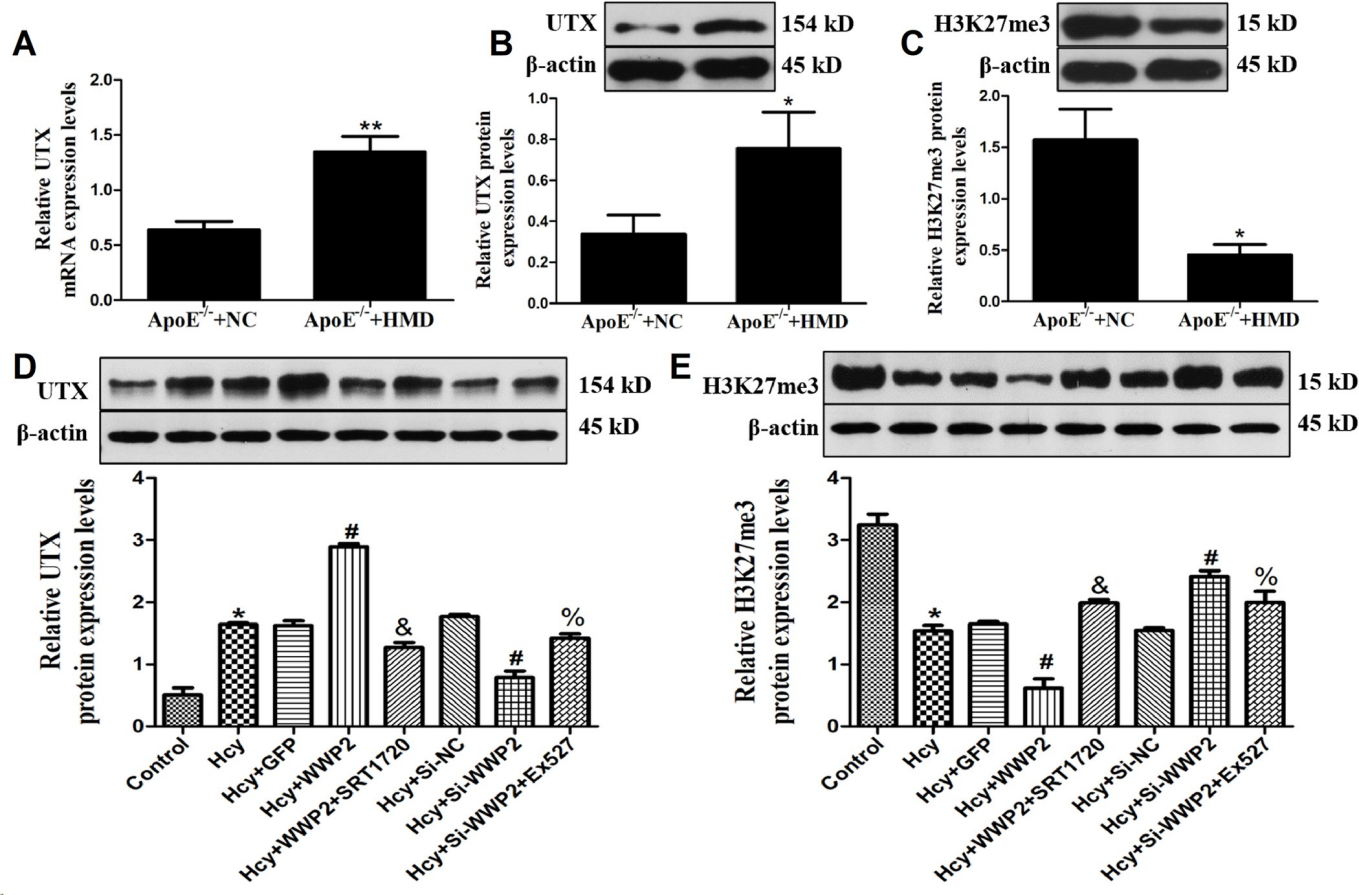
Hcy up-regulates WWP2 expression by inhibiting histone H3K27me3 via up-regulated UTX. A. UTX mRNA expression in mice aortic VSMC. B. UTX protein expression in mice aortic VSMC. C. H3K27me3 protein expression in mice aortic VSMC. D. UTX protein expression in Hcy-induced VSMC. E. H3K27me3 protein expression in Hcy-induced VSMC. The experiment was performed in triplicate, and the representative images are shown.*P<0.05 compared with the control group. ^**#**^*P*<0.05. compared with the Hcy group. ^%^*P*<0.05 compared with the Hcy+Si-wwp2 group. ^&^*P*<0.05 compared with the Hcy+WWP2 group.

To further investigate why WWP2 levels are elevated in hcy treated VSMC and mice. The mRNA and protein expression of histone demethylase (UTX) and the protein expression of histone H3K27me3 in aortic vessels of ApoE^-/-^ mice were detected (Fig 5A–5C). The results illustrated that UTX mRNA and protein expression were up-regulated and histone H3K27me3 protein expression was down-regulated in hcy treated VSMC and mice. *In vitro* experiments, it was found that after WWP2 overexpression, UTX expression was up-regulated, and H3K27me3 expression was down-regulated. At the same time of WWP2 overexpression, SIRT1 agonist SRT1720 interfered with VSMC, inhibited UTX expression and increased H3K27me3 expression. Furthermore, giving the SIRT1 inhibitor Ex527 to stimulate VSMC while knocking out WWP2 produced the opposite results (Fig 5D and 5E). These results suggested that UTX and histone H3K27me3 were involved in the up-regulation of WWP2 expression induced by Hcy, and Hcy might up-regulate WWP2 expression by inhibiting histone H3K27me3 through up-regulated UTX.

## Discussion

The current study revealed that Hcy regulated SIRT1/STAT3 phosphorylation via upregulating WWP2 to promote VSMC proliferation, migration and phenotypic switch, and the mechanism of upregulation of WWP2 by Hcy may be through upregulation of histone demethylase UTX and inhibition of histone H3K27me3.

VSMC proliferation, migration and phenotype switch are considered as significant factors for the development of AS [21]. Hcy, a non-protein, sulfur-containing amino acid formed exclusively via S-adenosylmethionine (SAM) demethylation, can induce the proliferation, migration and phenotypic transformation of VSMC, thus promoting the occurrence of AS. However, the particular mechanism underlying the development of AS remains unclear. Herein, VSMC were induced with Hcy. Consistent with previous reports [8], the results of the present study showed that cell treatment with Hcy enhanced the proliferation, migration and phenotypic transformation of VSMC. However, the particular mechanisms underlying the effects of Hcy on VSMC need to be further investigated.

VSMC and VSMC-derived cells play a crucial role in atherogenesis and the formation of atherosclerotic lesions. It has been reported that ⁓70% of the components in atherosclerotic plaques are VSMC and their derivatives [22], while the remaining 40% consist of foam cells [4, 23, 24]. VSMC are characterized by high plasticity and they can therefore reversibly switch between quiescent and active states. The phenotypic switch from a contractile to a synthetic state has been associated with several pathophysiological processes during AS [1]. A synthetic phenotype is commonly characterized by decreased expression of contractile proteins, such as a-SMA, and increased cell proliferation and migration rates [25]. Furthermore, a-SMA down-regulation during the early stages of AS has been associated with OPN, a secreted matricellular cytokine involved in cell adhesion, proliferation and migration. VSMC with synthetic phenotype typically express OPN, which acts as a critical regulator of proliferative cardiovascular diseases [26–29]. In the current study, the expression levels of OPN, α-SMA and SM22α in Hcy-treated VSMC or in ApoE^*-/-*^ mice fed with HMD were the same as those observed in previous studies and verified the transition of VSMC into a synthetic phenotype during the onset of AS.

WWP2, encoded by the WWP2 gene, is expressed in several tissues/organs throughout the human body and regulates several cellular, physiological and pathological processes [29, 30]. WWP2 was associated with oxidative stress-mediated VSMC injury in different cardiovascular diseases, such as AS, ischemia-reperfusion injury, cardiomyopathy and heart failure [31, 32], suggested that WWP2 could be a significant target for treating several cardiovascular diseases.

In the present study, a key role of WWP2 in Hcy-induced VSMC proliferation, migration and phenotypic transformation was revealed. WWP2 mRNA and protein levels are elevated in Hcy treated VSMC and mice. *In vitro*, WWP2 overexpression promoted the Hcy-induced proliferation, migration and phenotypic transformation of VSMC from a contractile to a synthetic phenotype. However, WWP2 silencing exhibited the opposite results, thus indicating that WWP2 could be involved in the Hcy-mediated proliferation, migration and phenotypic transformation of VSMC. Further study on the mechanism of Hcy induced WWP2 up-regulation found that UTX and histone H3K27me3 were involved in the up-regulation of WWP2 expression induced by Hcy, and Hcy might up-regulate WWP2 expression by inhibiting histone H3K27me3 through up-regulated UTX.

To the best of our knowledge the present study was the first to demonstrate that the expression levels of the key genes SIRT1 and STAT3 altered in the presence of Hcy. Therefore, SIRT1 was downregulated and p-STAT3 was upregulated in Hcy-treated VSMC. SIRT1, a protein encoded by the human SIRT1 gene, is a nicotinamide adenine dinucleotide (NAD+) dependent deacetylase [33]. It has been suggested that the histone deacetylase SIRT1 exhibits protective effects against arterial senescence and AS [34–38]. STAT3 is a transcription factor encoded by the human STAT3 gene. It has been reported that the acetylation and phosphorylation of STAT3 serve a crucial in the proliferation, migration and phenotypic transformation of VSMC [39]. Targeted inhibition of STAT3 could be a potential therapeutic strategy for AS [40]. It has been previously reported that STAT3 promotes VSMC proliferation and migration mainly through its phosphorylation activity. Compared with STAT3^+/+^ mice, STAT3^-/-^ mice exhibited significantly fewer phospholipid oxide-mediated atherosclerotic areas in the aortic roots. Furthermore, STAT3 gene knockout could significantly reduce AngII-induced VSMC proliferation and migration [41]. It was therefore hypothesized that WWP2 could play a significant role in the proliferation and migration of VSMC and angiogenesis, and this effect is related to the regulation of SIRT1/STAT3 by WWP2.

The above studies suggested that WWP2 could exhibit an interactive association with SIRT1/STAT3 and play a crucial role in the proliferation and migration of VSMC. However, it is not clear whether the above mechanism also underlies the Hcy-induced proliferation, migration and phenotypic transformation of VSMC. Here in, the protein expression levels of SIRT1 were decreased, while those of p-STAT3 was increased in Hcy treated VSMC and mice. Similar effects were observed in WWP2 overexpressing VSMC, since SIRT1 was also significantly downregulated and p-STAT3 was upregulated. The opposite results were obtained in WWP2-depleted VSMC. Furthermore, the results demonstrated that p-STAT3 was notably upregulated following cell treatment with a SIRT1 inhibitor and downregulated in VSMC treated with a SIRT1 stimulant. These findings indicated that SIRT1 and STAT3 could be involved in Hcy-induced VSMC injury, while WWP2 could regulate the expression of SIRT1 and p-STAT3. Additionally, the expression of p-STAT3 was significantly enhanced upon SIRT1 inhibition and reduced by SIRT1 stimulation, thus suggesting that SIRT1 could negatively regulate STAT3 expression in Hcy-induced VSMC.

## Conclusion

Our study provides a new evidence that Hcy regulated SIRT1/STAT3 phosphorylation via upregulating WWP2 to induce the proliferation, migration and phenotypic transformation of VSMC. WWP2 may be an important target for Hcy induction of AS.

## Supporting information

S1 Table Sequence information of primers used in RT-PCR. (DOCX)

S1 Raw images (PDF)
