# Supplementary material for: Proliferation, migration and phenotypic transformation of VSMC induced via Hcy related to up-expression of WWP2 and p-STAT3
Source: PLoS One. 2024 Jan 2;19(1):e0296359. doi: 10.1371/journal.pone.0296359 (PMC10760878; doi:10.1371/journal.pone.0296359)
Supplement: S1 Table — (DOCX) [file pone.0296359.s001.docx]

**Table 1** Sequence information of primers used in RT-PCR

| **Gene** | **Primers sequence** | **Tm(℃)** |
| --- | --- | --- |
| *WWP2* | F：5’-CAAAGCCCAAGGTGCATAATCG-3’ | 62 |
|  | R：5’-CCAATGCGCTTCCCAGTCT-3’ |  |
| *SIRT1* | F：5′-TGGACAATTCCAGCCATCTC-3′ | 60 |
|  | R：5′-GCGTGTCTATGTTCTGGGTATAG-3′ |  |
| *UTX* | F：5'-CACTCAGAGTCCACAAAG-3' | 59 |
|  | R：5'-GGCATCTTCTCTTCTTTG-3' |  |
| *α-SMA* | F：5’-GCCAAGACTGGGACCAGGAA-3’ | 59 |
|  | R：5’-TGGTTCTGACGCCAGTGGA-3’ |  |
| *SM22α* | F：5’-TGGCAGTGACCAAGAATGAT-3’ | 56 |
|  | R：5’-GGTCGTCCGTAGCCTGTC-3’ |  |
| *OPN* | F：5’-AGCGAGGAGTTGAATGGTGCATAC-3’ | 56 |
|  | R：5’-AATCTGGACTGCTTGTGGCTGTG-3’ |  |
| *GAPDH* | F：5’-CATCTCTGCCCCCTCTGCTGA-3’ | 56 |
|  | R：5’-GGATGACCTTGCCCACAGCCT-3’ |  |
