# Supplementary material for: Proliferation, migration and phenotypic transformation of VSMC induced via Hcy related to up-expression of WWP2 and p-STAT3
Source: PLoS One. 2024 Jan 2;19(1):e0296359. doi: 10.1371/journal.pone.0296359 (PMC10760878; doi:10.1371/journal.pone.0296359)
Supplement: S1 Raw images — (PDF) [file pone.0296359.s002.pdf]

The original images of all blot or gel results

Fig.1 H-J. Western blot analysis was used to detect the α-SMA, SM22a and OPN protein levels in ApoE$^{-/-}$ mice aortic VSMC.

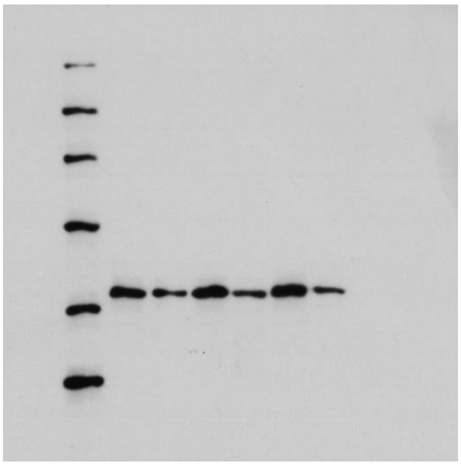

a-SMA

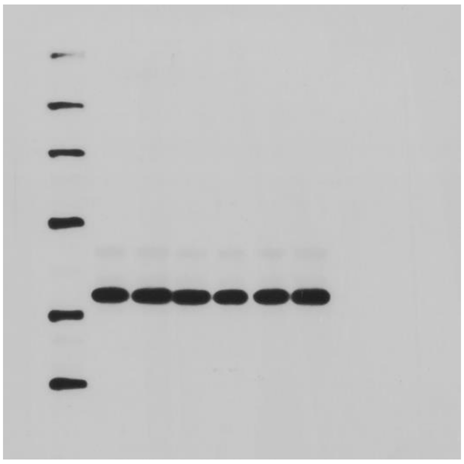

β-actin

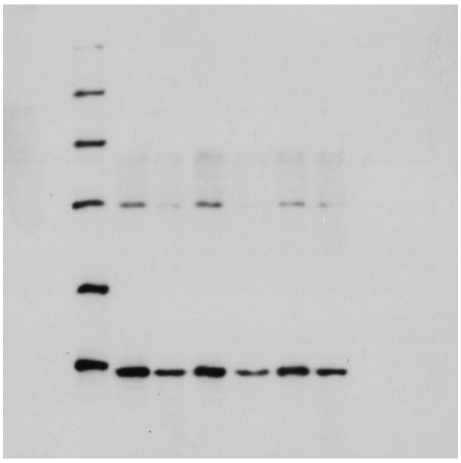

SM22a

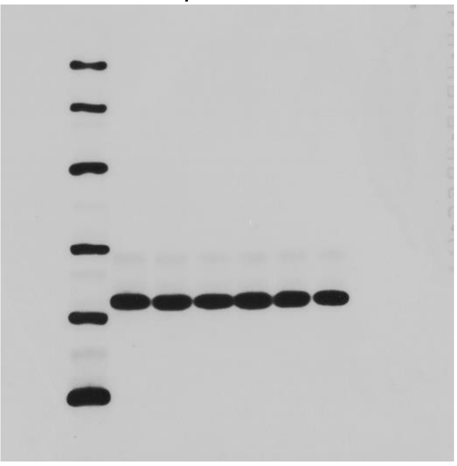

β-actin

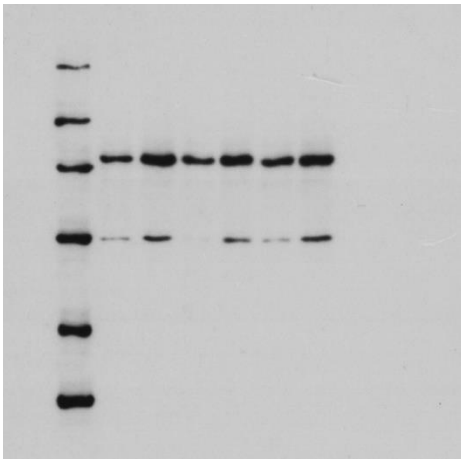

OPN

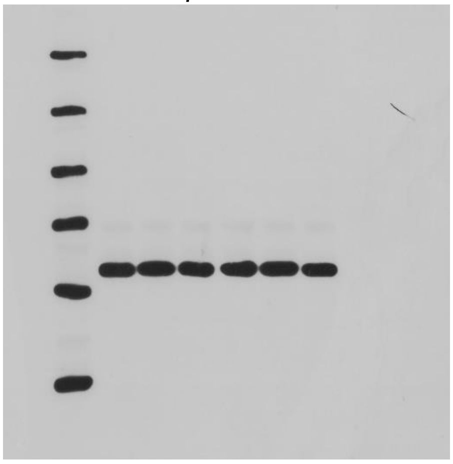

β-actin

The Original blots/gels are the Marker, ApoE$^{-/-}$ + NC group, and ApoE$^{-/-}$+HMD group. The experiment was performed in triplicate.

Fig.2 A. WWP2 protein expression in mice aortic VSMC.

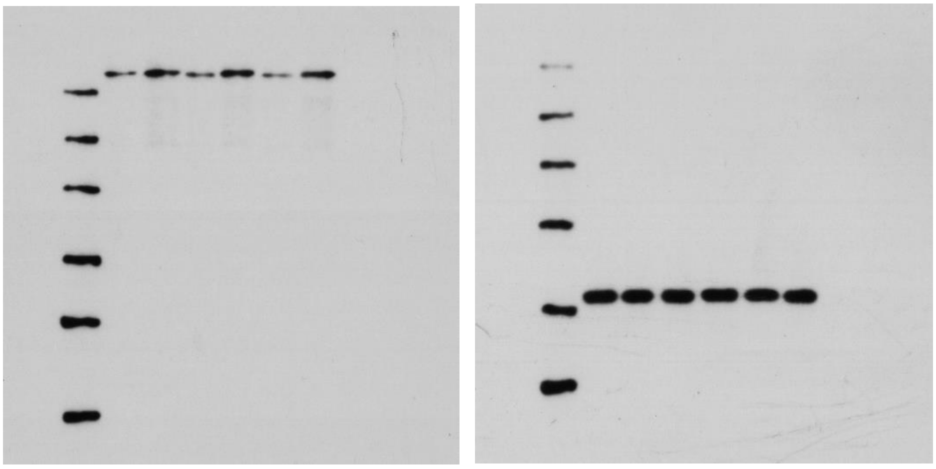

WWP2 β-actin

The Original blots/gels are the Marker, ApoE$^{-/-}$ + NC group, and ApoE$^{-/-}$+HMD group. The experiment was performed in triplicate.

B. WWP2 protein expression in VSMC.

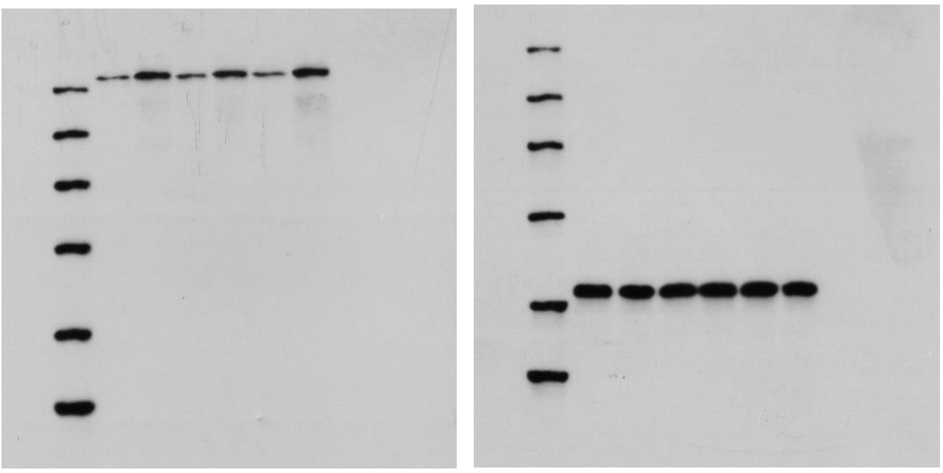

WWP2 β-actin

The Original blots/gels are the Marker, Control, and Hcy groups. The experiment was performed in triplicate.

Fig.3 A. The transfection efficiency of WWP2 overexpression and interference plasmid was determined by western blot.

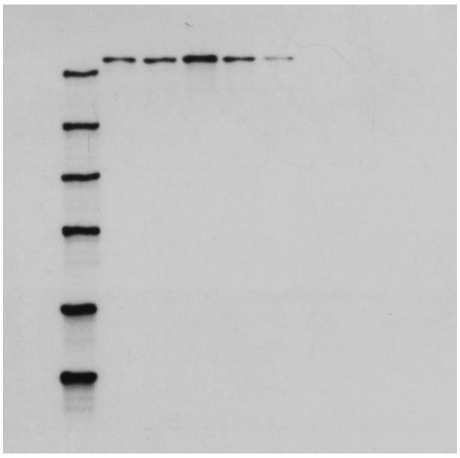
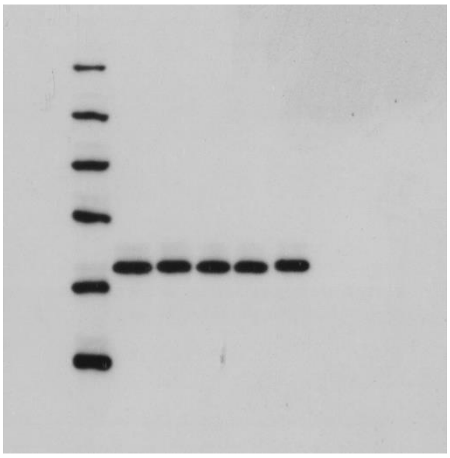

WWP2 β-actin

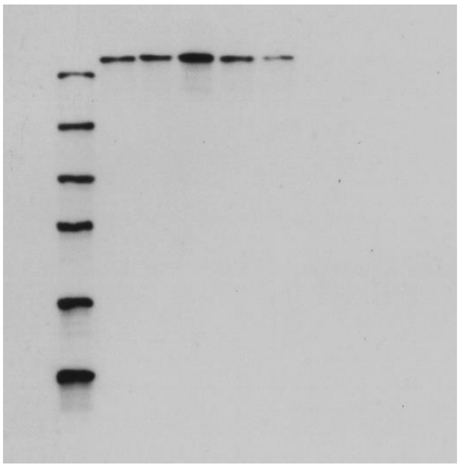
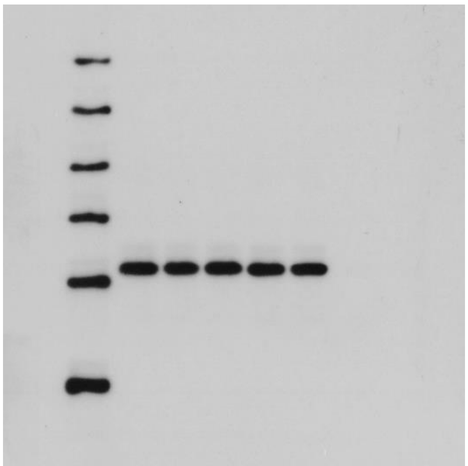

WWP2 β-actin

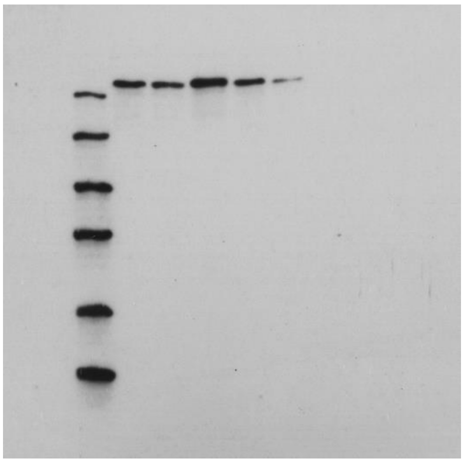
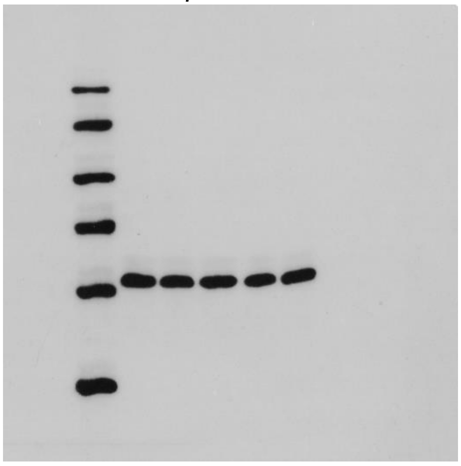

WWP2 β-actin

The Original blots/gels are the Marker, Control group, GFP group, WWP2 group, Si-NC group, and Si-WWP2 group. The experiment was performed in triplicate.

H. Western blot was used to detect a-SMA, SM22a, and OPN protein expressions.

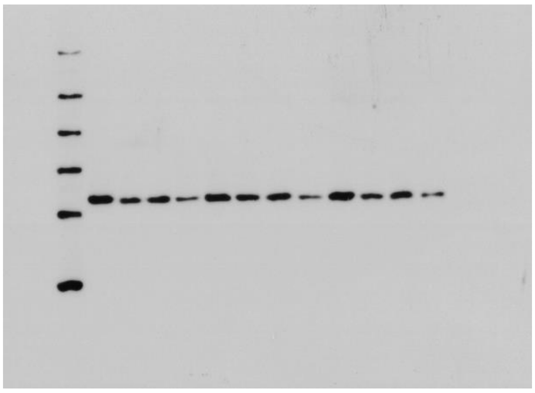

a-SMA

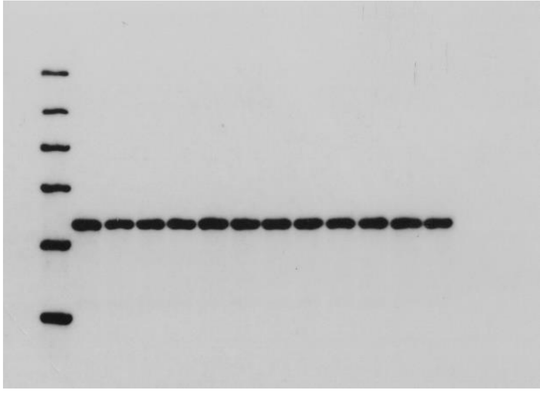

β-actin

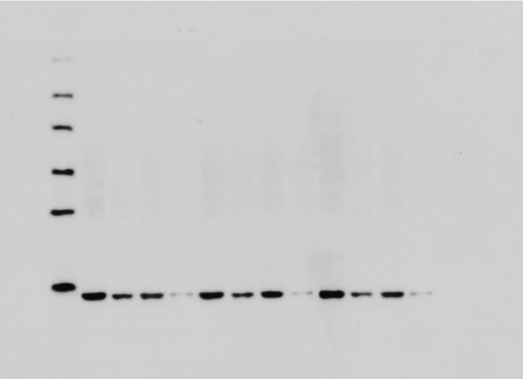

SM22a

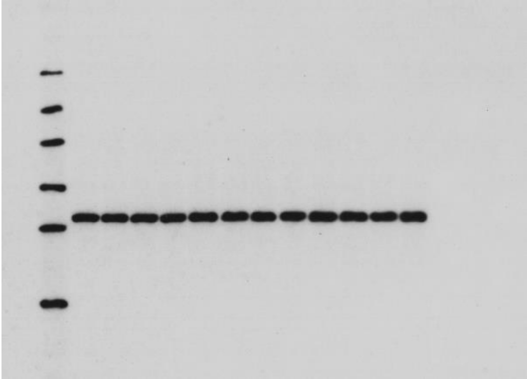

β-actin

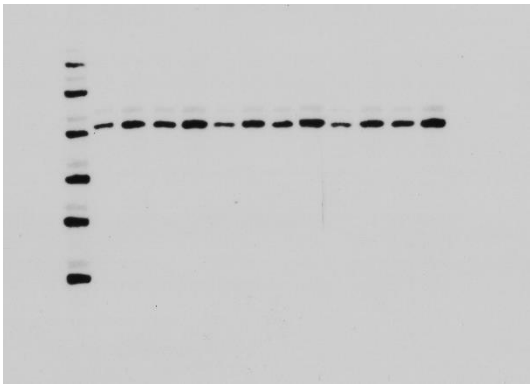

OPN

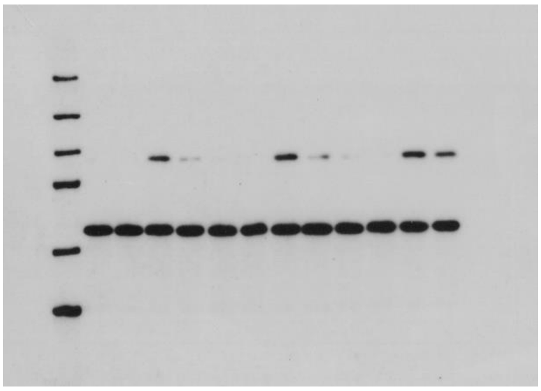

β-actin

The Original blots/gels are the Marker, Control group, Hcy group. Hcy+Si-WWP2 group, and Hcy+WWP2 group. The experiment was performed in triplicate.

Fig.4 D. SIRT1 protein expression in mice aortic VSMC.

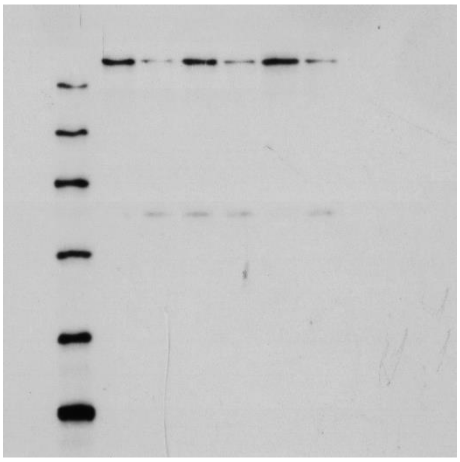
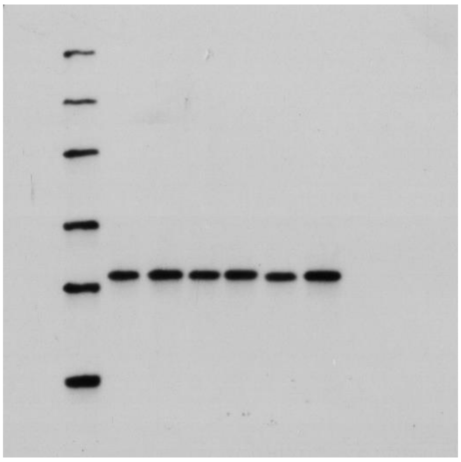

SIRT1 β-actin

The Original blots/gels are the Marker, ApoE$^{-/-}$ + NC group, and ApoE$^{-/-}$+HMD group. The experiment was performed in triplicate.

E. p-STAT3 protein expression in mice aortic VSMC.

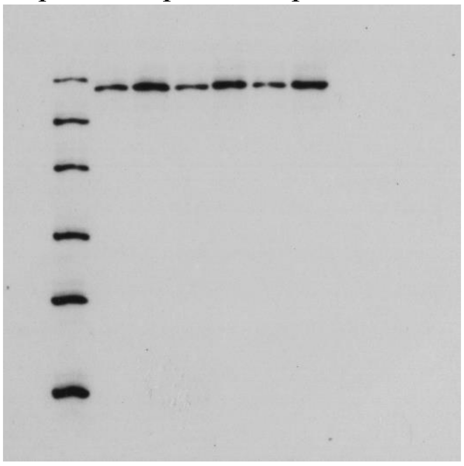
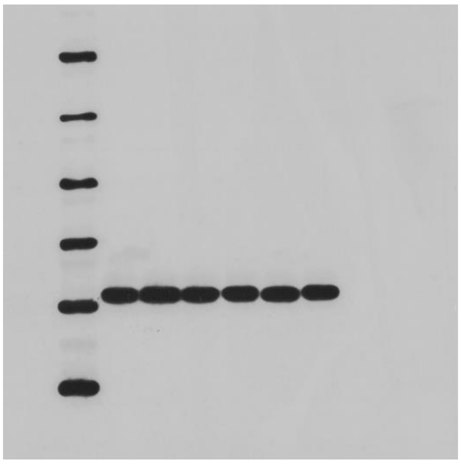

p-STAT3 β-actin

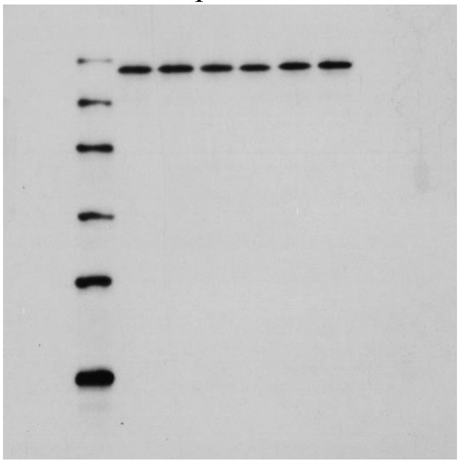
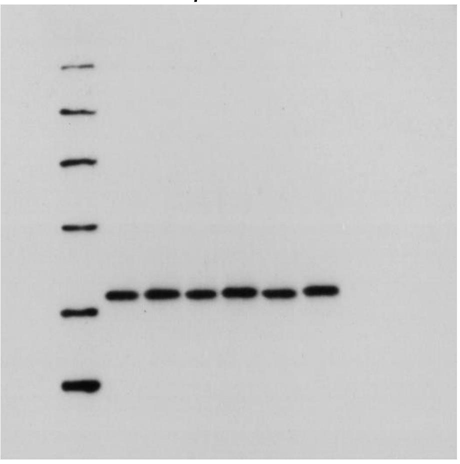

STAT3 β-actin

The Original blots/gels are the Marker, ApoE$^{-/-}$ + NC group, and ApoE$^{-/-}$+HMD group. The experiment was performed in triplicate.

F. SIRT1 protein expression in Hcy-induced VSMC.

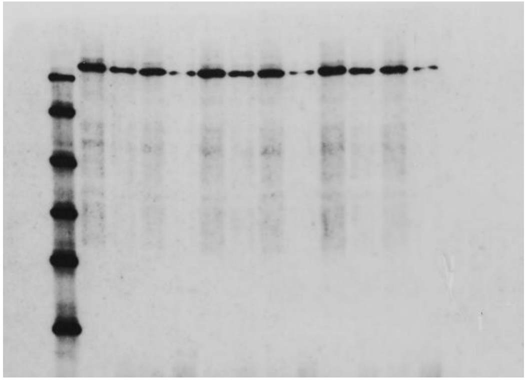

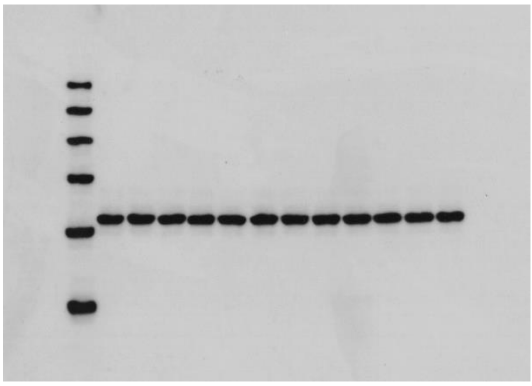

SIRT1 β-actin

The Original blots/gels are the Marker, Control group, Hcy group. Hcy+Si-WWP2 group, and Hcy+WWP2 group. The experiment was performed in triplicate.

G. p-STAT3 and STAT3 protein expression in Hcy-induced VSMC.

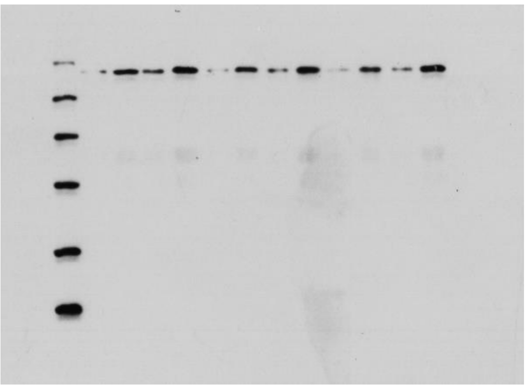

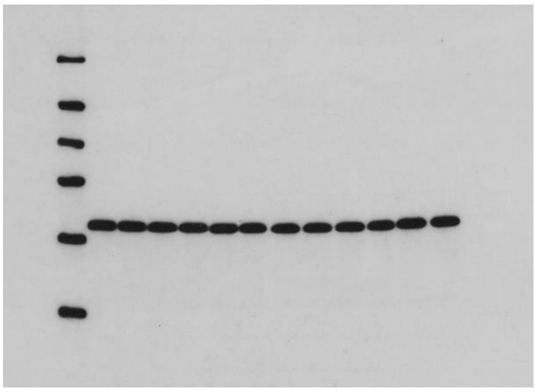

p-STAT3 β-actin

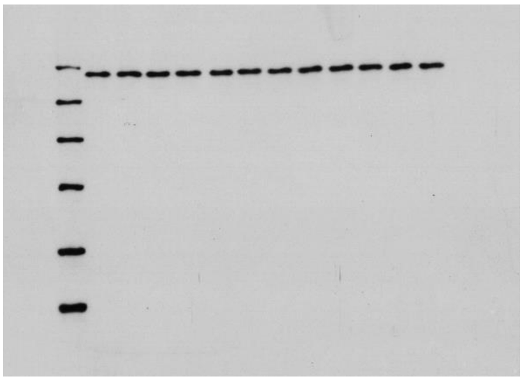

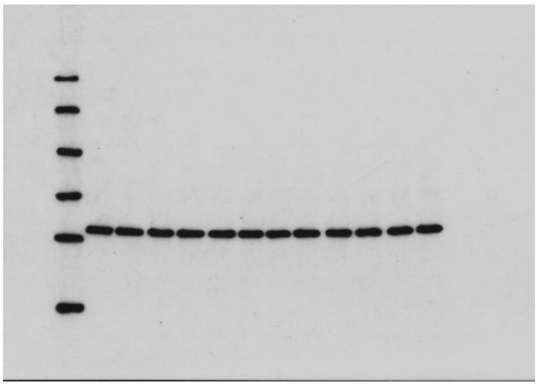

STAT3 β-actin

The Original blots/gels are the Marker, Control group, Hcy group. Hcy+Si-WWP2 group, and Hcy+WWP2 group. The experiment was performed in triplicate.

H. The effects of the Ex527 and SRT1720 on p-STAT3 and STAT3 protein expression in Hcy-induced VSMC.

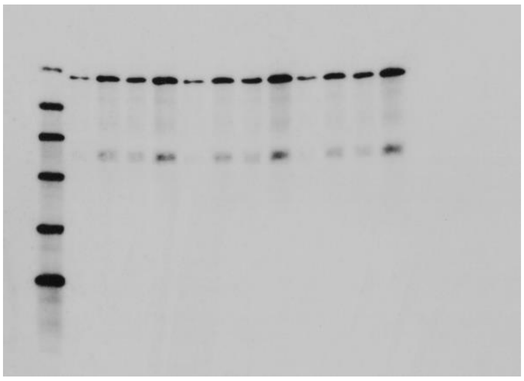

p-STAT3

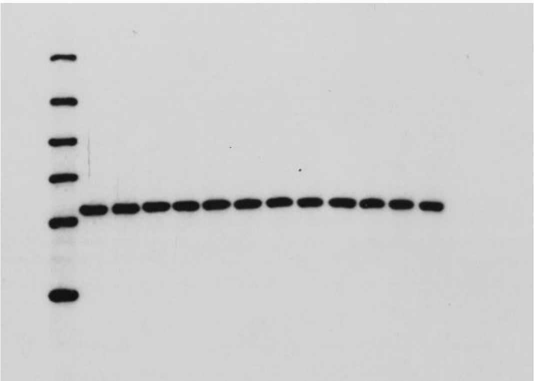

β-actin

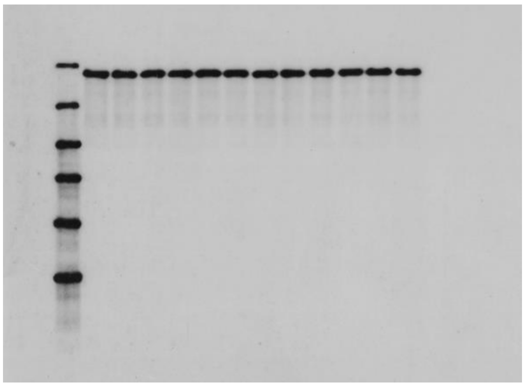

STAT3

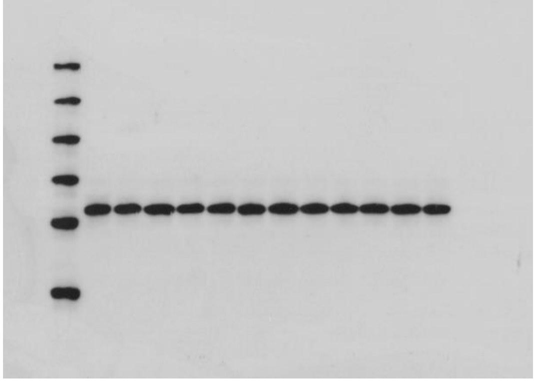

β-actin

The Original blots/gels are the Marker, Control group, Hcy group. Hcy+SRT1720 group, and Hcy+Ex527 group. The experiment was performed in triplicate.

Fig.5 B. UTX protein expression in mice aortic VSMC.

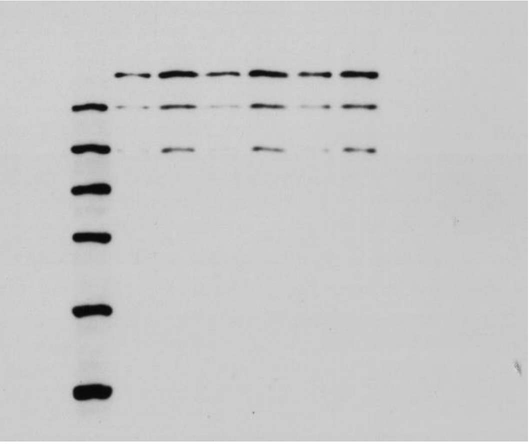

UTX

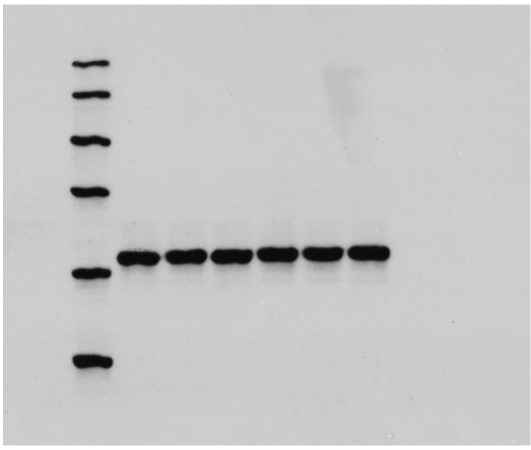

β-actin

The Original blots/gels are the Marker, $ApoE^{-/-}$ + NC group, and $ApoE^{-/-}$+HMD group. The experiment was performed in triplicate.

C. H3K27me3 protein expression in mice aortic VSMC.

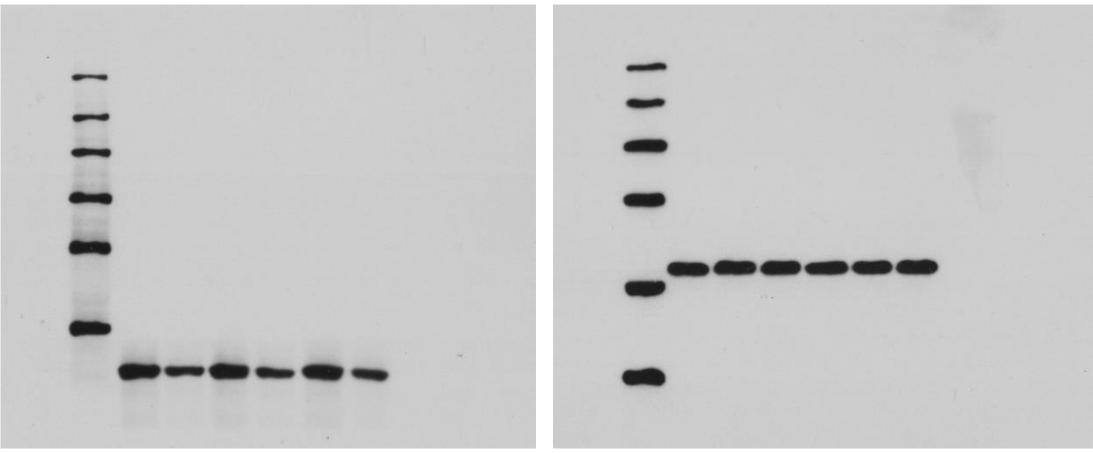

H3K27me3 β-actin

The Original blots/gels are the Marker, $ApoE^{-/-}$ + NC group, and $ApoE^{-/-}$+HMD group. The experiment was performed in triplicate.

D. UTX protein expression in Hcy-induced VSMC.

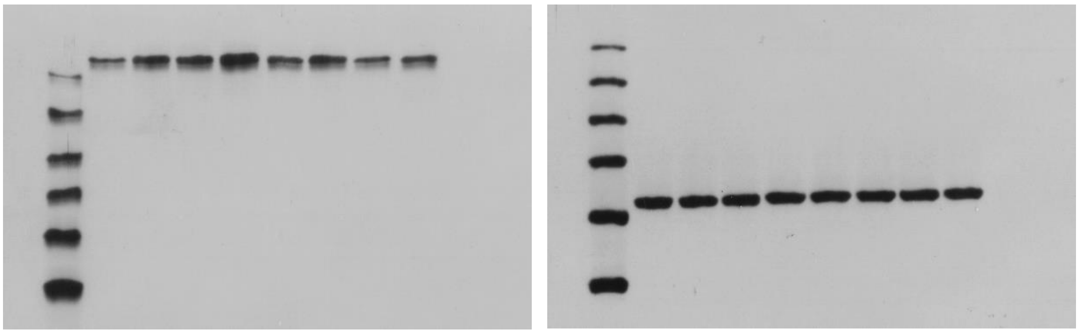

UTX β-actin

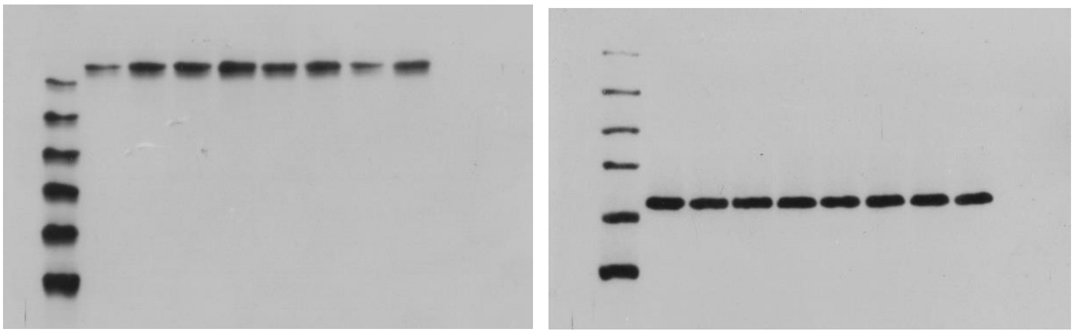

UTX β-actin

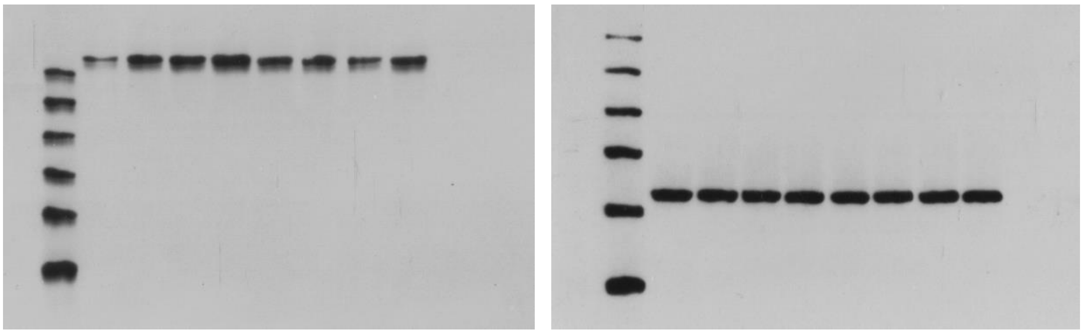

UTX β-actin

The Original blots/gels are the Marker, Control group, Hcy group, Hcy+GFP group, Hcy+WWP2 group, Hcy+WWP2+SRT1720 group, Hcy+Si-NC group, Hcy+Si-WWP2 group, and Hcy+Si-WWP2+Ex527 group. The experiment was performed in triplicate.

E. H3K27me3 protein expression in Hcy-induced VSMC.

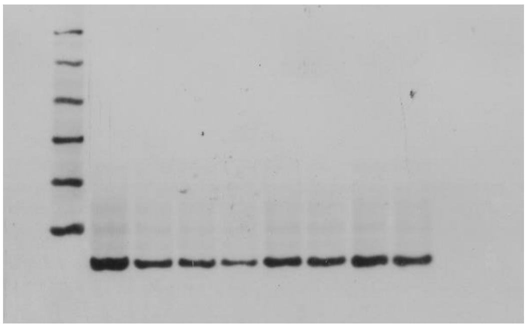

H3K27me3

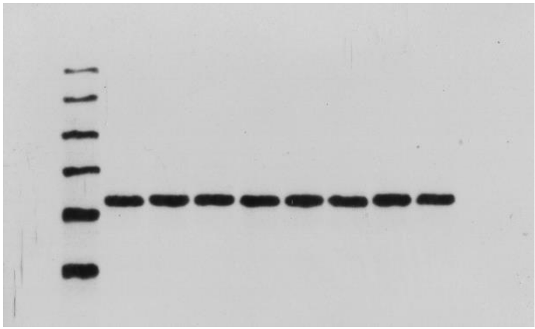

β-actin

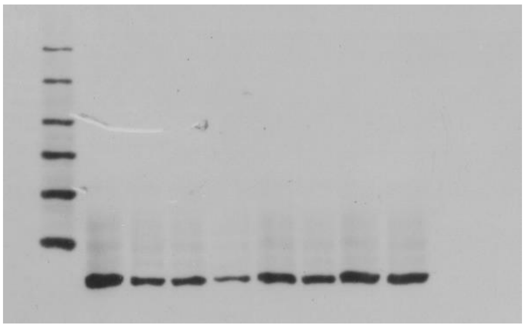

H3K27me3

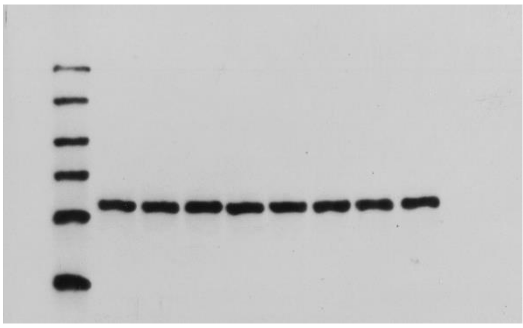

β-actin

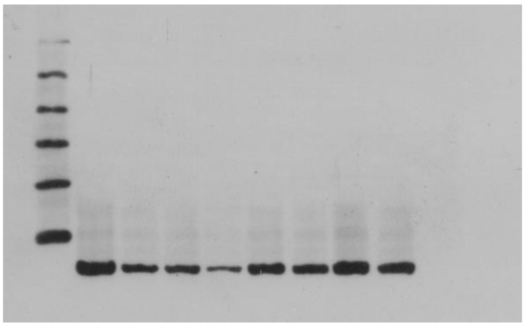

H3K27me3

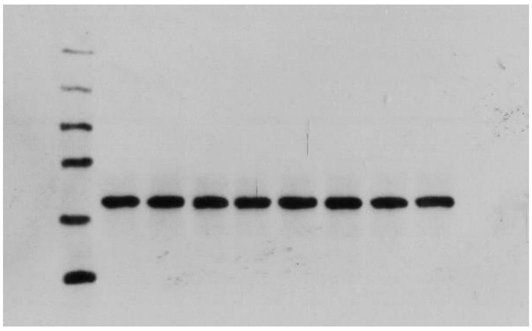

β-actin

The Original blots/gels are the Marker, Control group, Hcy group, Hcy+GFP group, Hcy+WWP2 group, Hcy+WWP2+SRT1720 group, Hcy+Si-NC group, Hcy+Si-WWP2 group, and Hcy+Si-WWP2+Ex527 group. The experiment was performed in triplicate.
